# Association of maternal primary language with neurodevelopmental outcomes in high-risk infants

**DOI:** 10.1038/s41372-025-02438-3

**Published:** 2025-09-29

**Authors:** Nicole Nghiem, Maria Martes Gomez, My H. Vu, Alexis Deavenport-Saman, Douglas Vanderbilt, Ashwini Lakshmanan, Sarah Wang, Ani Boodaghian, Christine B. Mirzaian

**Affiliations:** 1 Department of Pediatrics, Children’s Hospital Los Angeles, Los Angeles, CA USA; 2 Biostatistics Core, Children’s Hospital Los Angeles, Los Angeles, CA USA; 3 Keck School of Medicine, University of Southern California, Los Angeles, CA USA; 4 Department of Health Systems Science, Kaiser Permanente Bernard J. Tyson School of Medicine, Pasadena, CA USA

**Keywords:** Paediatrics, Health policy, Outcomes research, Risk factors

## Abstract

**Objective:**

Examine whether maternal primary language (English or a minoritized language) is associated with differences in infant developmental outcomes.

**Study design:**

This retrospective cohort study included 253 infants from a high-risk infant follow-up clinic at a quaternary care center in the U.S. who underwent developmental evaluation at 6, 12, and 24 months corrected age. The Bayley Scales, 3rd and 4th editions, were used to assess development.

**Result:**

At 6- and 12-month visits, the minoritized-language group had significantly lower scores than the English-language group in cognitive, language, and motor domains. By the 24-month visit, the minoritized-language group appeared to catch up in cognitive and motor domains but a significant difference in language skills persisted.

**Conclusion:**

Our findings indicate disparities in developmental outcomes, particularly in language skills at 24 months corrected age, based on maternal primary language, underscoring the need for further research to inform targeted interventions to address these disparities.

## Introduction

High-risk neonates have been found to be at increased risk for developmental delays and poor long-term educational and behavioral outcomes [1–4]. The high-risk infant population includes those with perinatal conditions that increase risk for developmental delay, including prematurity, special health care needs (e.g., congenital heart disease or central nervous system abnormalities), or dependence on durable medical equipment [5–7]. This population has been found to have greater academic difficulty and increased behavioral challenges, including higher rates of attention-deficit/hyperactivity disorder and autism spectrum disorder diagnoses [8–12].

Maternal primary language has not been well-studied in relation to developmental outcomes in the high-risk infant population and available findings are conflicting. One study found that extremely premature infants whose family’s primary language was Spanish had similar cognitive but lower language scores on developmental testing than children from primarily English-speaking homes while another study did not find an association between primary language and language scores [13, 14]. Seeking a better understanding of the association between maternal primary language and infant developmental outcomes becomes increasingly important as the immigrant population in the United States continues to grow with only about half of this population (54%) reporting English speaking proficiency on a 2022 immigration study [15]. A recent systemic review identified disparities in healthcare access and poorer health outcomes for individuals in the U.S. with limited English proficiency [16]. Another study found that children with autism spectrum disorder from families whose primary language was not English experienced greater barriers to accessing to services [17]. These studies highlight the barriers experienced by families who speak minoritized languages, limiting their access to services and impacting health outcomes. By examining the impact of maternal primary language on developmental outcomes in high-risk neonates, we can identify possible targets for future interventions to ameliorate disparities in care.

There is a paucity of research examining the association between primary language and developmental outcomes among high-risk infants. Thus, the purpose of our study was to assess the influence of maternal primary language—English compared to Spanish or another language other than English (subsequently referred to as minoritized language)—on short-term developmental outcomes of infants in the U.S. following NICU discharge. As an exploratory aim, we examined differences in developmental outcomes between low and high Child Opportunity Index (COI) level groups, a proxy for socioeconomic status based on geographic location.

We hypothesized that high-risk infants of minoritized-language mothers would have poorer developmental outcomes and trajectories than those of English-language mothers. For the exploratory aim, we postulated that the lower COI level group would have poorer developmental outcomes than those in the higher COI level group.

## Methods

### Data collection

We performed a retrospective cohort study of patients seen in high-risk infant follow-up (HRIF) clinic at a quaternary care center with initial visit (post-NICU discharge) between July 2016 and June 2019 and final visit (24 months corrected age) by December 2021. Infants were seen for developmental evaluation up to 3 times at 6, 12, and 24 months corrected age. Inclusion criteria: infants with gestational age <32 weeks, birth weight <1500 g or other eligible medical conditions per the California Children’s Services High Risk Infant Follow-up criteria, including central nervous system abnormalities (e.g., hypoxic ischemic encephalopathy, high-grade intraventricular hemorrhage, neonatal seizures), congenital heart disease, pulmonary conditions requiring supplemental oxygen, and systemic infections [5, 6]. Exclusion criteria: maternal language of preference was not noted or missing 6-month visit developmental evaluation score. Infant development was assessed using the cognitive, language, and motor composite scores on the Bayley Scales of Infant and Toddler Development (BSID)—3rd and 4th editions were used during the period of study and have been noted to be highly correlated [18, 19]. BSID assessments were performed by trained occupational and physical therapists assigned to the HRIF clinic. For families who preferred a language other than English, an interpreter was used. There was an in-person Spanish interpreter in the clinic that was utilized when available. Otherwise, a remote interpreter was used via video chat through a third-party professional service. Patient data abstracted from the electronic medical record included maternal primary language, BSID developmental scores, gestational age, birth weight, HRIF-eligible medical criteria, sex at birth, race/ethnicity, and address.

### Child opportunity index

As a proxy for socioeconomic status, we utilized the Childhood Opportunity Index (COI) 2.0. COI is a composite index that provides a multifaceted, intersectional approach to measuring opportunity for children based on geographic location. It is a validated, nationally normed, publicly available index based on census tract data. It is composed of 29 indicators that include education, health/environment, and social/economic variables, taking into account commonly studied variables such as high school graduation rate, health insurance coverage, and household income as well as more unique variables such as quality of early childhood education centers, access to green space, and exposure to pollutants. [20] COI level based on census tract data and nationally normed for 2017 was generated by inputting patients’ addresses into the COI maps webpage [21]. COI is stratified into five levels with each representing 20% of the United States child population—Very low (scores at or below the 20th percentile), Low (scores between the 20th–40th percentile), Moderate (scores between the 40th and 60th percentile), High (scores between the 60th and 80th percentile), Very high (scores above the 80th percentile) [20].

### Statistical analyses

Demographics and patient characteristics were summarized using mean and (SD) for continuous variables, and frequency and percentage for categorical variables. Differences between groups were assessed via Wilcoxon rank-sum tests and Fisher’s exact tests, as appropriate. The primary analyses utilized Wilcoxon rank-sum tests, as univariate analyses, and linear mixed-effects models with a random participant effect, to account for within-participant correlation, using R package “lmerTest” to test whether developmental scores were different between English- and minoritized-language groups at the 6-, 12-, and 24-month visits [22]. Models were adjusted for sex at birth, COI, and gestational age as we recognized the potential confounding effects of sex, COI, and gestational age on the observed relationships between English- and minoritized-language groups and developmental outcomes based on literature and clinical perspective. The secondary analyses examined the significance of Visit*Group interaction terms from the models above to assess whether the changes in developmental scores from the 6-month visit to the 12-month visit, and from the 6-month visit to the 24-month visit, were different between English- and minoritized-language groups.

A similar exploratory analysis was performed to assess whether developmental scores were different between families with lower and higher COI at the 6-, 12-, and 24-month visits. Due to low sample sizes in each COI stratification level, Very low/Low/Moderate groups were combined into a “lower” COI level group and High/Very high were combined into a “higher” group. Models were similar to the primary analyses.

All participants with developmental scores at least at the 6-month visit were included in analyses as mixed effects models are generally robust for unbalanced data across study time points [23]. In an additional analysis, there were no differences in patient characteristics, such as language, gestational age, and birth weight between patients with and without any follow-up data. Statistical significance was defined using a two-sided *p* < 0.05. All statistical analyses were conducted in R Studio 4.2.2.

## Results

### Demographics and characteristics

Of the 253 families included in this analysis, 180 had English as their language of preference and 73 (66 of which preferred Spanish) had a language of preference other than English. There were no significant differences between the two groups in terms of gestational age and sex at birth. Compared to the English-language group, a greater percentage of those in the minoritized-language group were Hispanic, publicly insured and had a lower COI level. Detailed results are presented in Table 1.

**Table 1:** Patient demographics and characteristics.

	Overall, *N* = 253^a^	English language of preference, *N* = 180^a^	Minoritized language of preference, *N* = 73^a^	*p* value^b^
Mean gestational age (weeks)	32.9 (5.4)	32.8 (5.6)	33.2 (5)	0.7
*Range*	23.0, 42.0	23.0, 42.0	23.0, 41.0	
Gestational age <32 weeks	103 (40.7%)	78 (43.3%)	25 (34.2%)	0.7
Mean birthweight (grams)	2 092.9 (1 098.4)	2 078.3 (1 110.9)	2 127.9 (1 074.6)	0.8
*Range*	500.0, 5 318.0	500.0, 4 950.0	500.0, 5 318.0	
(*Missing)*	*14*	*11*	*3*	
Sex at birth				0.9
Male	149 (59%)	107 (59%)	42 (58%)	
Female	104 (41%)	73 (41%)	31 (42%)	
Race/Ethnicity				<0.001
White	49 (19.7%)	49 (27.8%)	0 (0%)	
Hispanic	156 (62.7%)	91 (51.7%)	65 (89%)	
Black	14 (5.6%)	13 (7.4%)	1 (1.4%)	
Asian/Pacific Islander	26 (10.4%)	19 (10.8%)	7 (9.6%)	
Mixed Race	4 (1.6%)	4 (2.3%)	0 (0%)	
(*Missing)*	*4*	*4*	*0*	
Insurance				<0.001
Public	193 (76.6%)	122 (67.8%)	71 (98.6%)	
Private	59 (23.4%)	58 (32.2%)	1 (1.4%)	
(*Missing)*	*1*	*0*	*1*	
Child Opportunity Index level				0.004
Very low/Low/Moderate	201 (79.8%)	134 (74.9%)	67 (91.8%)	
High/Very high	51 (20.2%)	45 (25.1%)	6 (8.2%)	
(*Missing)*	*1*	*1*	*0*	

^a^Mean (SD); n (%).

^b^Wilcoxon rank sum test; Chi-square test.

Follow-up rates at 24 months compared to 6 months were 51% for the minoritized language group and 37% for the English-language group. Loss to follow-up was examined at 12 and 24 months with comparison of language, gestational age, and birth weight between those who followed up and those who did not; no significant difference was found. Besides prematurity, the most prevalent HRIF-eligible criteria among this population were chronic lung disease (CLD) requiring oxygen for >28 days (*n* = 58 (23%)), evidence of intracranial pathology or central nervous system problem associated with adverse neurological outcome (*n* = 58 (23%)), and other problems that could result in neurologic abnormality including CNS infection, sepsis, cardiovascular instability, bilirubin in excess of exchange transfusion level (*n* = 81 (32%)). Limited analysis of this data suggested higher rates of CLD with prolonged oxygen requirement (minoritized *n* = 24 (32%) vs English *n* = 34 (19%)) and other problems that could result in neurologic abnormality for the minoritized cohort (minoritized *n* = 33 (45%) vs English *n* = 48 (27%)) and higher rates of seizures for the English cohort (English *n* = 16 (8.9%) vs minoritized *n* = 0 (0%)). The significance of these findings cannot be determined due to our small data sample and the inconsistent data patterns observed.

### Differences in developmental outcomes

All univariate and multivariate analysis estimates are presented in Table 2. Minoritized-language infants, on average, had significantly lower cognitive scores than English-language infants at the 6-month and 12-month visits (mean [SD]: 89.45 [18.17] vs. 94.31 [15.41], and 84.39 [17.43] vs. 93.56 [16.82], respectively). This relationship remained significant in the multivariate model after adjusting for COI, sex at birth, and gestational age (*p* = 0.028 and 0.008). There was no longer a significant difference in cognitive scores between the two groups at the 24-month visit (*p* = 0.4). Similarly, motor scores were significantly lower in the minoritized-language group at the 6-month and 12-month visits unadjusted (mean [SD]: 80.51 [21.37] vs. 86.88 [17.73], and 77.09 [18.27] vs. 87.13 [17.83], respectively) and adjusted (*p* = 0.02 for both). There was no longer a significant difference in motor scores between the two groups at the 24-month visit (*p* = 0.7). Language scores were significantly lower in the minoritized-language group at all three timepoints compared to the English-language group (mean [SD]: 83.59 [16.14] vs 88.46 [13.83], 75.16 [12.26] vs 83.58 [15.65], and 71.97 [14.81] vs 80.40 [18.79], respectively), which was also observed in the multivariate model (*p* < 0.05).

**Table 2:** Mixed effect model results of Bayley Scales of Infant Development assessment scores at each timepoint comparing differences between English- and minoritized-language groups.

		Unadjusted				Adjusted	
	Timepoint	Overall^a^	English^a^	Minoritized^a^	*p* value^b^	Estimate (95% CI)^c^	*p* value
Cognitive	6-month	*N *= 253; 92.91 (16.37)	*N* = 180; 94.31 (15.41)	*N* = 73; 89.45 (18.17)	0.045	–5.2 (–9.8, –0.56)	0.028
	12-month	*N* = 184; 90.72 (17.49)	*N* = 127; 93.56 (16.82)	*N* = 57; 84.39 (17.43)	<0.001	–6.8 (–12, –1.8)	0.008
	24-month	*N* = 103; 84.59 (16.47)	*N* = 66; 85.88 (15.93)	*N* = 37; 82.30 (17.38)	0.3	–2.7 (–8.6, 3.2)	0.4
Language	6-month	*N* = 250; 87.04 (14.38)	*N* = 177; 88.46 (13.38)	*N* = 73; 83.59 (16.14)	0.023	–4.6 (–8.8, –0.44)	0.03
	12-month	*N* = 178; 80.98 (15.16)	*N* = 123; 83.58 (15.65)	*N* = 55; 75.16 (12.26)	<0.001	–7.1 (–12, –2.4)	0.003
	24-month	*N* = 90; 77.59 (17.93)	*N* = 60; 80.40 (18.79)	*N* = 30; 71.97 (14.81)	0.037	–7.6 (–14, –1.4)	0.016
Motor	6-month	*N* = 246; 85.07 (19.01)	*N* = 176; 86.88 (17.73)	*N* = 70; 80.51 (21.37)	0.028	–6.2 (–11, –1.0)	0.020
	12-month	*N* = 181; 83.97 (18.52)	*N* = 124; 87.13 (17.83)	*N* = 57; 77.09 (18.27)	<0.001	–6.7 (–12, –1.2)	0.018
	24-month	*N* = 102; 80.65 (17.26)	*N* = 65; 81.94 (17.17)	*N* = 37; 78.38 (17.41)	0.3	–1.3 (–7.7, 5.2)	0.7

Unadjusted results are reported as well as results adjusted for Child Opportunity Index, sex at birth, and gestational age.

^a^Sample size; Mean (SD).

^b^Wilcoxon rank sum test.

^c^CI = Confidence Interval; English = reference group.

### Differences in developmental trajectory

**Fig. 1:**
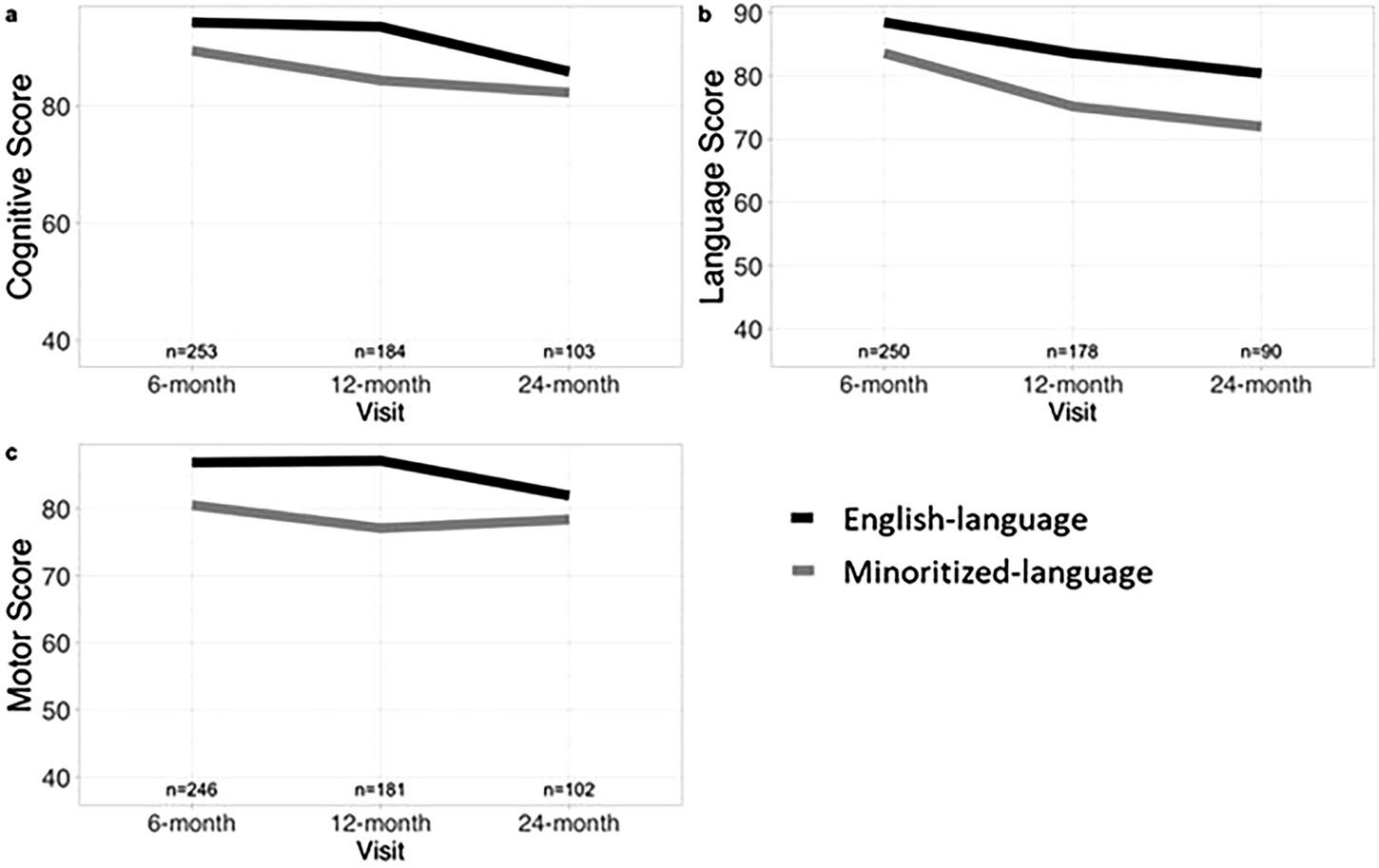
Developmental trends by language group. Developmental trends in each domain (panel **a**: cognitive, **b**: language, **c**: motor) for English- and minoritized-language groups from the 6-month to 12-month to 24-month visits.

When evaluating change from baseline (score at the 6-month visit) to the 12-month visit and to the 24-month visit, both minoritized- and English-language groups showed negative trends in all three domains as seen in Table 3 and Fig. 1. Minoritized-language infants’ cognitive scores decreased by −3.76 (95% CI: −7.4, −0.1) at the 12-month visit, and −8.03 (95% CI: −12.3, −3.8) at the 24-month visit. English-language infants had a smaller decrease of −2.11 (95% CI: −4.5, 0.33) at the 12-month visit and a larger decrease of −10.5 (95% CI: −13.6, −7.3) at the 24-month visit. However, the differences in these changes were not statistically significant between the two groups (−1.64 [95% CI: −6.0, 2.7]; *p* = 0.46 and 2.46 [95% CI: −2.9, 7.8]; *p* = 0.37). Although there were larger decreases in language scores for minoritized-language infants compared to the English-language infants (−7.76 [95% CI: −11.8, −3.7] and −5.46 [95% CI: −8.2, −2.7] at the 12-month visit, and −11.93 [95% CI: −17.1, −6.8] and −9.21 [95% CI: −12.8, −5.6] at the 24-month visit), the differences were not statistically significant (−2.3 [95% CI: −7.2, 2.6]; *p* = 0.36 and −2.71 [95% CI: −9, 3.6]; *p* = 0.4, respectively). For motor scores, there was no significant difference in the change in motor score between the minoritized- and English-language groups at the 12-month visit (−0.53 [95% CI: −5.3, 4.2]; *p* = 0.825). At the 24-month visit, English-language infants had a larger decrease of −7.15 (95% CI: −10.5, −3.8) in motor scores compared to −2.25 (95% CI: −6.8, 2,3) in the minoritized-language infants, but this difference was not significant (4.9 [95% CI: −0.8, 10.6]; *p* = 0.093).

**Table 3:** Comparison of developmental trajectory between English- and minoritized-language groups from the 6-month visit to the 12-month visit and from the 6-month visit to the 24-month visit.

		English group		Minoritized group		Minoritized—English	
		Estimate (95% CI)	*p*	Estimate (95% CI)	*p*	Estimate (95% CI)	*p*
Cognitive	12-month – 6–month	–2.11 (–4.5, 0.33)	0.09	–3.76 (–7.4, –0.1)	0.044	–1.64 (–6, 2.7)	0.462
	24-month – 6–month	–10.5 (–13.6, –7.3)	<0.001	–8.03 (–12.3, –3.8)	<0.001	2.46 (–2.9, 7.8)	0.368
Language	12-month – 6–month	–5.46 (–8.2, –2.7)	<0.001	–7.76 (–11.8, –3.7)	<0.001	–2.3 (–7.2, 2.6)	0.359
	24-month – 6–month	-9.21 (–12.8, -5.6)	<0.001	–11.93 (–17.1, –6.8)	<0.001	–2.71 (–9, 3.6)	0.4
Motor	12-month – 6–month	–1.76 (–4.4, 0.9)	0.189	–2.29 (–6.2, 1.6)	0.252	–0.53 (–5.3, 4.2)	0.825
	24-month – 6–month	–7.15 (–10.5, –3.8)	<0.001	–2.25 (–6.8, 2.3)	0.337	4.9 (–0.8, 10.6)	0.093

### COI and differences in developmental outcomes

In both univariate and multivariate analyses, there were no differences between lower and higher COI level groups for cognitive and motor scores at all timepoints (*p* > 0.05). The only exception was language score at the 24-month visit. Infants in the higher COI level group had significantly higher language scores than lower COI level group infants at the 24-month visit (mean [SD]: 86.16 [21.27] vs. 75.10 [16.38]; *p* = 0.028). This relationship remained significant (*p* = 0.01) in the multivariate model after adjusting for sex at birth and gestational age (model-estimate mean [95% CI]: 9.2 [2.1, 16.0]).

## Discussion

In our study of 253 high-risk neonates, we identified disparities in developmental outcomes with a widening gap in language skills by 24 months corrected age for the minoritized-language infant group. Interestingly, our exploratory analysis revealed that infants in the lower COI level group had significantly lower language scores at the 24-month visit compared to infants in the higher COI level group. This finding parallels the gap in language scores between minoritized- and English- language groups and highlights the connection between language-access and opportunity. Reasons for the disparities found between minoritized- and English-language families in this study may include barriers to accessing care and lack of language concordance between provider and patient [17, 24–28]. In addition, lower neighborhood opportunity level may have further impacted disparities in language outcomes as indicated by the results of our exploratory analysis. Minoritized-language families experiencing lower socioeconomic opportunity may face compounding inequities in care. Our findings that lower COI was associated with lower language scores at 24 months corrected age aligns with studies showing an association between lower socioeconomic status and decreased language enrichment [29].

Two studies of high-risk populations, one examining mother’s milk provision to NICU infants and one examining access to services for children with autism, found that parents speaking a minoritized language faced greater challenges in accessing support services and therapies [17, 24]. Parents who do not speak the majority language may be afraid to raise concerns and pursue evaluations and services due to factors such as lack of knowledge and resources, lack of empowerment, community mental health stigma, and worry about burden on the family [25, 26]. Barriers to logistical factors such as scheduling appointments and arranging transportation also prevent connection to care [25]. These factors may impact follow-up care and connection to early intervention services, which are correlated with positive health and developmental outcomes for this population [30, 31].

In addition, service quality and outcomes may be impacted by patient-provider racial, cultural, and language concordance [28, 32, 33]. A global dataset analysis revealed that being from a minority language-speaking household was associated with lower odds of on-track developmental scores compared to being from majority language-speaking households [27]. Other studies have shown that lack of patient-provider racial and language concordance and associated sense of connection and safety may affect rapport, engagement, and performance on assessments [25, 28, 33]. Families speaking a minoritized language have also perceived interpretation services to be inadequate due to limited availability of in-person interpreters or insufficient training of interpreters available in the medical settings [25]. Another consideration is whether the differences in language scores between children of majority and minoritized language speakers are truly a reflection of disparities in outcomes or rather a reflection of bias introduced by performing English language-based testing through an interpreter [14]. In addition, assessment of health literacy using validated tools can also be helpful in identifying additional communication needs [34]. These issues highlight the importance of staffing diverse, multi-lingual providers and providing access to appropriate supports, including reliable interpreters who can navigate both linguistic and cultural divides [32, 33].

Our study had several strengths. First, we were able to examine our cohort through longitudinal time points from NICU discharge to 24 months of age corrected. This allowed us to monitor the progression of developmental outcomes and make comparisons over time. Next, for COI, we used address rather than zip code data. Zip codes, designed by the United States Postal Service for efficient mail delivery routes, have been found to be less reliable than geocoded data and tend to overestimate neighborhood opportunity for Black and Hispanic children while underestimating neighborhood opportunity for White children [35, 36]. Lastly, despite a significant difference in COI levels between minoritized- and English-language groups, differences in developmental outcomes persisted when the sample was adjusted for COI, strengthening the association between maternal primary language and infant developmental outcomes.

There were several limitations to our study. One was loss to follow-up at subsequent visits. Between 6- and 24-month visits, the minoritized-language group surprisingly had a higher follow-up rate compared to the English-language group. Ideally, the follow-up rate would have been measured from NICU referral if that were available. Factors found to be associated with loss to HRIF follow-up include barriers such as lower maternal education level, greater distance from clinic, non-English primary language, Black non-Hispanic race, very low neighborhood opportunity level as well as indicators of lower medical severity such as shorter length of hospitalization and higher gestational age [37–39]. While we did not find a difference in language, gestational age, and birth weight for those who followed up and those that did not, there may be undetected confounding effects from individual socioeconomic factors that were unavailable in our chart review. We used COI as a proxy for socioeconomic status, but it is determined by one’s address at a single point in time and may not reflect long-term opportunity. A family’s opportunity level may fluctuate over time and can be influenced by individual factors such as household income and time in poverty [40]. Another limitation was missing birth and NICU hospitalization data, often from outside referrals, limiting our ability to utilize neonatal medical severity indices, which would have allowed us to more precisely compare medical severity between the minoritized- and English-language groups [41]. We were at least able to correct for gestational age, which has been strongly correlated with infant morbidity and mortality [42]. Next, although our therapists performing BSID assessments were highly experienced clinically, we could not ensure interrater reliability, given that this was a retrospective study so there may have been variation between assessors. Additionally, interpreters were used for non-English assessments, which added additional time to the assessment and may have altered the standardization of administration. Lastly, the Bayley kits used were in English and consideration for impact of language and cultural differences on developmental testing outcomes is important in the interpretation of these results [33].

Our findings demonstrate disparities in developmental outcomes, particularly in language skills, for infants of mothers who speak Spanish or another minoritized language in the United States. While this group appeared to catch up to their English-language counterparts in the cognitive and motor domains by 24 months corrected age, a difference in language domain scores persisted. Few studies have examined disparities correlated with maternal primary language. These findings underscore the need for further research to inform targeted interventions to address disparities in developmental testing and outcomes associated with minoritized primary language and low socioeconomic opportunity. Addressing health inequity is an important goal for neonatal care, which does not end at discharge from the NICU.

## Data Availability

The datasets analyzed for this study are available from the corresponding author on reasonable request.
